# Personality and Risk of Frailty: the English Longitudinal Study of Ageing

**DOI:** 10.1007/s12160-016-9833-5

**Published:** 2016-09-22

**Authors:** Catharine R Gale, René Mõttus, Ian J Deary, Cyrus Cooper, Avan Aihie Sayer

**Affiliations:** 1 MRC Lifecourse Epidemiology Unit, Southampton General Hospital, University of Southampton, Southampton, SO16 6YD UK; 2 Centre for Cognitive Ageing & Cognitive Epidemiology, Department of Psychology, University of Edinburgh, Edinburgh, UK; 3 Department of Psychology, University of Tartu, Tartu, Estonia

**Keywords:** Personality, Frailty, Prospective study, Ageing

## Abstract

**Background:**

There is evidence that the personality traits conscientiousness, extraversion and neuroticism are associated with health behaviours and with risk of various health outcomes. We hypothesised that people who are lower in conscientiousness or extraversion or higher in neuroticism may be at greater risk of frailty in later life.

**Methods:**

We used general linear models to examine the prospective relation between personality, assessed using the Midlife Development Inventory, and change in frailty, modelled by a frailty index, in 5314 men and women aged 60 to over 90 years from the English Longitudinal Study of Ageing.

**Results:**

Men and women with higher levels of neuroticism or lower levels of extraversion or conscientiousness had an increased frailty index score at follow-up. After adjustment for potential confounding or mediating variables, including frailty index score at baseline, the frailty index score at follow-up—which potentially ranges from 0 to 1—was higher by 0.035 (95 % confidence interval 0.018, 0.052) for a standard deviation increase in neuroticism and lower by 0.061 (0.031, 0.091) or 0.045 (0.020, 0.071) for a standard deviation increase in extraversion or conscientiousness, respectively. There was some evidence that the association between extraversion and frailty may be due to reverse causation whereby poorer health affected responses to items in the personality inventory.

**Conclusions:**

Higher levels of neuroticism or lower levels of conscientiousness or extraversion may be risk factors for the onset or progression of frailty. Future studies need to replicate these observations in other populations and explore the mechanisms underlying these associations.

## Introduction

Frailty is a clinical syndrome observed in older people whose core feature is an increased vulnerability to stressors due to impairments in multiple, inter-related systems, decreased physiological reserves and a decline in the ability to maintain homeostasis [1]. It is increasingly common at older ages [2] and raises the risk of numerous adverse consequences, including disability, falls, morbidity, hospitalisation, institutionalisation and death. Its causes are complex and are likely to involve not only just biomedical but also social and psychological mechanisms [3].

There are two established models for frailty [1]. The frailty phenotype model—devised by Fried and colleagues using data from the Cardiovascular Health Study [4]—defines frailty as the presence of three or more components: unintentional weight loss, self-reported exhaustion, low-energy expenditure, slow walking speed and weak grip strength. The frailty index, or cumulative deficit model—originally developed by Rockwood and colleagues using data from the Canadian Study of Health and Aging [5, 6]—defines frailty in terms of the accumulation of ‘deficits’ (symptoms, signs, diseases and disabilities), whereby an individual’s frailty index score reflects the proportion of potential deficits present in that individual and indicates the likelihood that frailty is present [7]. While both models of frailty have predictive validity for adverse outcomes, there is evidence that the continuously distributed frailty index may be better at discriminating between moderately and severely frail individuals [8].

One psychological factor that may influence the risk of becoming frail in later life is personality—a largely stable set of traits and characteristics that influence behaviour, thoughts and feelings. Personality starts developing during early childhood and demonstrates increasing continuity with increasing age [9]. It has the potential to influence health via several mechanisms [10], including engagement in health-damaging or health-enhancing behaviours, illness behaviour in response to symptom perception or diagnosis of illness, or susceptibility to stress-induced physiological arousal, which may contribute to the development or progression of illness. There is evidence that levels of personality traits may change in adulthood in some people, if not in most, possibly in response to biological or environmental factors [11–13]. Such changes have been linked with later adverse health outcomes, including mortality [14]. This raises the possibility that personality traits themselves could, in the future, be targets for intervention.

The five-factor model of personality [15], consisting of the major personality traits of neuroticism, extraversion, openness to experience, agreeableness and conscientiousness, has been widely used to study the relationship between personality and health outcomes and behaviours [16]. To our knowledge, no previous study has investigated the relationship between these personality traits and frailty, but there is evidence that certain personality traits are associated with health behaviours or conditions that have been linked with risk of frailty, including smoking [17], physical activity [18], cardiovascular disease [19, 20], diabetes [21, 22] obesity [23, 24] and poorer cognitive function [25, 26]. Of the five major traits, conscientiousness—the tendency to be organised, responsible, industrious and disciplined—is the personality trait that has been most consistently linked with healthier behaviour [27] and with longevity [28–33]. People who are higher in conscientiousness also have a reduced risk of dying from cardiovascular disease [34–36]; are less likely to develop or die of diabetes [37]; and have a lower risk of obesity [38], cognitive decline [39] and dementia [40]. Neuroticism—the tendency to experience negative emotions—has been linked with smoking and low physical activity [41]. People who are higher in neuroticism have an increased risk of cognitive decline [39] and dementia [40], but findings on their risk of cardiovascular disease have been mixed, with some studies finding an increased risk [34, 42] and others finding no association [28, 43]. Extraversion—the tendency to be sociable, outgoing and energetic—has consistently been associated with being less physically active [41]. These findings on personality led us to hypothesise that older people who are lower in conscientiousness or extraversion or higher in neuroticism may be at greater risk of frailty. We had no a priori hypotheses about the relationship between the other major personality traits, agreeableness—the tendency to be kind, warm, tolerant and affable—and openness to experience—the tendency to be curious, creative, open to new ideas and intellectual—and risk of frailty.

The English Longitudinal Study of Ageing is a large population-based study of older men and women. We used these data to investigate the prospective relationship between the five major personality traits and frailty in people aged 60 to over 90 years.

## Methods

### Participants

The data for this study come from the English Longitudinal Study of Ageing (ELSA). The initial sample for ELSA was based on people aged ≥50 years who had participated in the Health Survey for England in 1998, 1999 or 2001 [44]. It was drawn by postcode sector and stratified by health authority and proportion of households in non-manual socioeconomic groups. The initial survey took place in 2002–2003. Subsequent waves of data collection have taken place at 2-year intervals. Refreshment samples drawn from the Health Survey for England were added at waves 3 and 4 to maintain the representation of people aged 50–75. The current study uses data from waves 5 (2010–2011) and 6 (2012–2013). Ethical approval was obtained from the Multicentre Research and Ethics Committee. Participants gave written informed consent.

### Measures

#### Frailty

We used a frailty index to assess frailty status at baseline (wave 5) and follow-up (wave 6). A frailty index can be derived from different numbers or types of variables, thereby facilitating comparison between datasets [7]. The criteria for inclusion are that the variables are associated with health status, represent conditions that become more common with age—though not ubiquitous (e.g. presbyopia)—and cover a range of systems [45]. If a frailty index is to be used at two or more time points on the same individuals, the items used to derive the index at each point in time need to be the same [45]. In ELSA, our frailty index was made up of 44 deficits, including sensory and functional impairments, a score on a composite measure of cognitive function that was in the lowest 10 % of the distribution, and self-reported comorbidities (see Supplementary Table 1 for details of the deficits included). The frailty index is constructed by summing the number of deficits present for each individual and dividing by the total number of deficits considered, which gives a range from 0 to 1. Higher values indicate greater frailty.

**Table 1:** Baseline characteristics of the study sample and their rank order correlations with frailty index scores at follow-up (*n* = 5314)

Baseline characteristics	Mean (SD), median (IQR) or number (%)	Correlation with frailty index at follow-up
Age (years), mean (SD)	70.0 (7.36)	0.3012***
Female, no. (%)	2982 (54.4)	0.128***
Personality traits, mean (SD)		
Extraversion	3.15 (0.55)	−0.249***
Agreeableness	3.51 (0.48)	0.015
Openness	2.86 (0.56)	−0.165***
Neuroticism	2.06 (0.58)	0.161***
Conscientiousness	3.27 (0.50)	−0.224***
Household wealth (£), median (IQR)	241,102 (131,215–418,219)	−0.292***
Smoking status, no. (%)		0.103***
Never	1956 (36.8)	
Ex-smoker	2822 (53.1)	
Current smoker	536 (10.1)	
Physical activity, no. (%)		−0.415***
Sedentary	279 (5.25)	
Low	1294 (24.4)	
Medium	2749 (51.7)	
High	992 (18.7)	
Frailty index score, median (IQR)	0.068 (0.022–0.136)	0.830***

***p < 0.001, Spearman correlation significance level

#### Personality

Levels of the five major personality traits—extraversion, agreeableness, conscientiousness, neuroticism and openness to experience—were assessed at wave 5 using a version of the Midlife Development Inventory previously used in the US Health and Retirement Survey [46]. These dimensions were measured using self-ratings of 26 adjectives. Respondents were asked the degree to which each adjective described them, rating each one on a four-point Likert scale (ranging from 1 to 4). The adjectives making up each dimension were as follows: extraversion: outgoing, friendly, active, talkative and lively; agreeableness: warm, helpful, soft-hearted, sympathetic and caring; conscientiousness: organised, responsible, thorough, hardworking and careless; neuroticism: moody, worrying, nervous and calm; and openness to experience: creative, imaginative, intelligent, curious, sophisticated and adventurous. Each score was calculated by obtaining the average of the ratings defining that dimension. Cronbach alpha values in these data were 0.76 (extraversion), 0.80 (agreeableness), 0.68 (neuroticism), 0.67 (conscientiousness) and 0.79 (openness to experience), indicating at least adequate internal consistency.

#### Covariates

We chose age, socioeconomic position, smoking and physical activity, all measured at baseline, as covariates. Socioeconomic position was indexed by total household wealth, including savings and investments, value of any property or business assets and net of debt, excluding pension assets. Household wealth has been identified as the most accurate indicator of long-term socioeconomic circumstances in ELSA [47]. Participants provided information on whether they were current smokers, were ex-smokers or had never smoked. Participants were asked about the level of physical activity involved in their job (if they were working) and responded to three questions on mild, moderate or vigorous physical activity carried out in daily life. The answers to these questions were used to derive a categorical summary variable on physical activity (sedentary, low, moderate or high) that approximates as closely as possible to the classification used in the Allied Dunbar Survey of Fitness [48].

#### Analytical Sample

In total, 7122 cohort members aged 60 and over took part in the baseline survey at wave 5. The current analysis is based on 5314 (75 %) of them who had complete data on personality, frailty index score and all the covariates at baseline and frailty index score at the wave 6 follow-up.

#### Statistical Analysis

We used rank order correlations to examine baseline characteristics in relation to the frailty index scores at follow-up. Frailty index scores have a gamma distribution [45], so we used general linear models assuming a gamma distribution to examine the relation between a standard deviation increase in each personality trait at baseline and frailty index score at follow up. Preliminary analyses showed that associations between personality traits and frailty index score did not differ by sex, so we analysed men and women together. We adjusted for age, sex and baseline frailty index score, then in addition for the other personality traits and next for household wealth, smoking status and physical activity Analyses were carried out using STATA version 13 (StataCorp 2013, College Station, TX). We used the STATA command ‘mfpigen’ to investigate whether the effect of personality traits on frailty index scores varied significantly according to the covariates. Mfpigen investigates interactions between pairs of variables, while simultaneously applying multivariable functional polynomials to the remaining variables to select a ‘confounder model’, which is used to adjust the interaction model for possible confounding by other covariates [49, 50]. In view of the large sample size and the likelihood that even small effects would be statistically significant, we used *p* < 0.01 to indicate statistical significance.

All data were weighted to correct for sampling probabilities, non-response and differential sample loss between waves in order to make them more closely reflect the population from whom the ELSA sample was drawn. Detailed descriptions of these weights and their calculation can be found in the technical reports on the study available at www.ifs.org.uk/elsa.

## Results

Table 1 shows the baseline characteristics of the 5314 men and women in the study and the rank order correlations between those characteristics and the frailty index scores at follow-up. Greater frailty at follow-up, as indicated by a higher frailty index score, was associated with older age, being female, lower household wealth, greater exposure to smoking, lower physical activity, lower extraversion, lower conscientiousness, lower openness, higher neuroticism and a higher frailty index at baseline. There was no association between baseline levels of agreeableness and frailty index score at follow-up. People who were excluded from our analytical sample due to loss to follow-up or missing data were, on average, older (mean 73.8 vs 70.0 years), frailer (median frailty index score 0.114 vs 0.068), poorer (median household wealth £188,900 vs £241,142), less physically active (22.6 % sedentary vs 5.25) and more likely to be a current smoker (14.2 vs 10.1 %). Those excluded had slightly lower scores for conscientiousness (mean 3.18 vs 3.27), extraversion (mean 3.07 vs 3.14) and openness (2.79 vs 2.86). There were no differences between our analytical sample and those excluded from it as regard sex distribution or scores for neuroticism and agreeableness.

The personality traits agreeableness, openness, extraversion and conscientiousness were all moderately positively correlated with each other (rho 0.41 to 0.59); correlations between these four traits and neuroticism were weaker and inverse (rho −0.04 to −0.20).

Table 2 shows mean (SD) personality trait scores and median (IQR) frailty index scores by age group in men and women separately. In both sexes, mean scores for the personality traits were lower with increasing age; the only exception was agreeableness in men where mean scores were slightly higher in older age groups. Mean differences in personality between the youngest and oldest age groups were small in size in both sexes: Cohen’s *d* = 0.2–0.3. In both sexes, median frailty index scores at baseline and at follow-up were higher and more dispersed with increasing age.

**Table 2:** Personality trait scores and frailty index scores according to age group in men and women

	Men			Women		
	Age group			Age group		
	60–69 (*n* = 1255)	70–79 (*n* = 827)	≥80 (*n* = 266)	60–69 (*n* = 1558)	70–79 (*n* = 1025)	≥80 (*n* = 383)
Personality traits, mean (SD)						
Extraversion	3.12 (0.56)	3.11 (0.56)	2.95 (0.57)	3.25 (0.51)	3.16 (0.56)	3.02 (0.60)
Agreeableness	3.39 (0.51)	3.40 (0.50)	3.40 (0.51)	3.62 (0.41)	3.59 (0.45)	3.51 (0.51)
Openness	2.96 (0.52)	2.87 (0.54)	2.76 (0.54)	2.89 (0.55)	2.80 (0.57)	2.69 (0.60)
Neuroticism	2.06 (0.59)	1.95 (0.57)	1.94 (0.53)	2.16 (0.58)	2.06 (0.57)	1.97 (0.53)
Conscientiousness	3.28 (0.50)	3.19 (0.50)	3.12 (0.50)	3.38 (0.45)	3.25 (0.49)	3.13 (0.55)
Frailty index score at baseline, median (IQR)	0.045 (0.023–0.091)	0.068 (0.023–0.136)	0.113 (0.068–0.227)	0.068 (0.023–0.114)	0.091 (0.045–0.182)	0.159 (0.091–0.273)
Frailty index score at follow-up, median (IQR)	0.045 (0.023–0.113)	0.068 (0.023–0.136)	0.136 (0.068–0.250)	0.068 (0.023–0.136)	0.114 (0.045–0.205	0.182 (0.091–0.295)

In both men and women, all personality traits, with the exception of agreeableness in men, and both frailty index scores differed significantly by age group (*p* < 0.001)

Table 3 shows the results of generalised linear models estimating the relation of personality traits at baseline (per standard deviation increase in score) with change in frailty index scores by follow-up. In model 1, where we examined each personality trait separately and adjusted for age, sex and baseline frailty index score, people who were more extravert, more conscientious and lower in neuroticism at baseline had a lower frailty index score at follow-up. There were no associations between baseline agreeableness or openness and frailty index score at follow-up. In model 2, where we further adjusted for all personality traits simultaneously, greater extraversion, greater conscientiousness and lower neuroticism continued to be significantly associated with lower frailty index scores at follow-up; adjusting for other personality traits strengthened the size of the effects between extraversion and conscientiousness and frailty index score at follow-up. In this model, unexpectedly, a significant association emerged between greater agreeableness at baseline and higher frailty index score at follow-up. In the final model, we further adjusted for household wealth, physical activity and smoking status at baseline. This additional adjustment resulted in only slight changes to the estimates. Greater extraversion, greater conscientiousness and lower neuroticism continued to be significantly associated with lower frailty index scores at follow-up. Greater agreeableness continued to be associated with a higher frailty index at follow-up in this multivariate adjusted model. We examined whether associations between personality traits and frailty index scores at follow-up varied according to the covariates. There was only one significant interaction, between conscientiousness and smoking status (*p* < 0.001): the association between conscientiousness and frailty index score was present and similar in non-smokers and current smokers but was absent in ex-smokers.

**Table 3:** Coefficients (95 % CI) for the effects of a standard deviation increase in personality trait scores at baseline on change in frailty index scores by follow-up

Personality trait scores, per SD	Coefficient (95 % CI)		
	Model 1	Model 2	Model 3
Agreeableness	0.011 (−0.010, 0.032)	0.072 (0.045, 0.100)***	0.04658 (0.031, 0.085)***
Openness	−0.023 (−0.044, −0.002)	0.013 (−0.014, 0.041)	0.019 (−0.008, 0.046)
Extraversion	−0.049 (−0.071, −0.027)***	−0.072 (−0.10, 2–0.033)***	−0.061 (−0.091, −0.031)***
Neuroticism	0.045 (0.023, 0.066)***	0.032 (0.015, 0.041)**	0.035 (0.018, 0.052)***
Conscientiousness	−0.052 (−0.074, −0.03)***	−0.072 (−0.102, −0.041)***	−0.045 (−0.071, −0.020)***

Model 1 adjusts for age, sex and frailty index score at baseline. Model 2 further adjusts for all personality trait scores at baseline. Model 3 further adjusts for household wealth, physical activity and smoking status at baseline

***p < 0.001, **p < 0.01: general linear model significance levels

In the analyses described above, our adjustment for baseline frailty index score reduces the possibility of reverse causation whereby poorer health at the time that the personality inventory was completed might have influenced how participants responded to some of the items in the inventory. Examination of the rank order correlations between the individual items making up the personality inventory and frailty index score at baseline showed that the extraversion items ‘active’ and ‘lively’ and the conscientiousness item ‘hardworking’ were much more strongly correlated with the contemporaneous frailty index score than the other items, with figures for rho of −0.46, −0.29 and −0.26, respectively (see Supplementary Table 2). As a further check on whether reverse causation might explain our findings, we carried out a sensitivity analysis. Firstly, we repeated our analyses looking at the relations between personality trait scores and later frailty index score in a subset of 3089 participants whose score on the baseline frailty index—potential range 0 to 1—was <0.08, in other words, those who were in generally good health with no or very few problems. In this subset of largely healthy individuals, the fully adjusted (model 3) associations between baseline personality traits and frailty index score at follow-up remained significant and were very similar in size to those observed in the sample as a whole. Secondly, we repeated our analyses of the relations between extraversion and conscientiousness in the whole sample using a modified score for each trait, which had been calculated without using the extraversion items active and lively or the conscientiousness item hardworking (the Cronbach alpha for these two modified scores showed acceptable internal consistency at 0.68 and 0.62, respectively). In a fully adjusted model using these modified scores, greater conscientiousness continued to be a significant predictor of a lower frailty index score at follow-up, but the association between extraversion and frailty index score at follow-up was no longer significant (*p* = 0.51).

## Discussion

In this prospective study of people aged 60 to over 90 years, higher levels of neuroticism and lower levels of extraversion and conscientiousness were associated with greater frailty at follow-up around 2 years later. These associations persisted after adjustment for several potential confounding or mediating variables. Results of sensitivity analyses suggested that the association between extraversion and frailty may be due to reverse causation whereby poorer health at baseline may have influenced responses to specific items used to assess extraversion. Unexpectedly, higher levels of agreeableness were also associated with greater frailty at follow-up, though these associations only emerged when we controlled for the variance agreeableness shared with other personality traits. We examined whether the associations between personality traits and frailty index score varied according to levels of the covariates, but there were no statistically significant interactions with the exception of one between conscientiousness and smoking status that was hard to interpret. In view of the large number of potential interactions examined, it is possible that this interaction was statistically significant by chance.

The finding that older people who were higher in neuroticism or lower in extraversion or conscientiousness scored higher on a frailty index at follow-up after adjustment for frailty level at baseline and other potential covariates provides support for our hypothesis that these traits may be risk factors for onset or worsening of frailty. To our knowledge, there have been no previous studies into the relation between personality and risk of frailty, either modelled using a frailty index, as here, or as the Fried phenotype. But, there is some evidence to link personality with key components of the Fried frailty phenotype. For example, evidence from the Baltimore Longitudinal Study of Ageing suggests that muscle strength is poorer in those who are higher in neuroticism or lower in extraversion [51]. Similarly, in a prospective study of older people in Chicago, higher neuroticism and lower extraversion were associated with more rapid decline in motor function [52], while in the Health, Aging, and Body Composition Study, lower conscientiousness was associated with slower walking speed and greater decline in walking speed over a 3-year follow-up period [53]. The mechanisms underlying these associations between personality and components of the frailty phenotype are not fully understood. While lifestyle factors and disease status partially explained the cross-sectional associations between neuroticism, extraversion or conscientiousness and muscle strength [51] or walking speed [53] and the longitudinal association between neuroticism and extraversion and decline in motor function [52], they appeared to play no part in the longitudinal relation between conscientiousness and decline in walking speed [53]. This is consistent with other studies that have found that the protective effect of higher conscientiousness on mortality from all causes or cardiovascular disease was only partially explained by health behaviours, obesity or other common risk factors such as diabetes and hypertension [28, 34, 35]. In the current study too, the associations that we found between conscientiousness, neuroticism and extraversion and risk of frailty as measured by a frailty index were only partially attenuated by adjustment for potential mediating factors—smoking and physical activity—and potential confounders. Further studies are needed using other personality inventories to try to replicate our findings and to explore whether it is specific lower-order facets of these personality traits that influence frailty risk.

It is possible that physiological mechanisms underlie links between personality and later frailty. There is evidence that personality is associated with individual differences in physiological processes that have been hypothesised to underlie the onset of frailty [54], namely inflammation [55, 56] and the hypothalamic-pituitary-adrenal axis (HPA) [57]. Some longitudinal studies have found that risk of frailty is increased in older people with higher blood concentrations of inflammatory markers, but associations are not consistent [21, 58–60]. Recent findings suggest that frailty is accompanied by blunted cortisol reactivity, but the direction of effect in this cross-sectional survey is unclear [61]. Further longitudinal studies are needed to explore the extent to which inflammation or HPA dysregulation explains links between neuroticism, extraversion or conscientiousness and risk of frailty.

The observation in the current study that being higher in agreeableness became a risk factor for greater frailty at follow-up once we controlled for levels of other personality traits was unexpected. We are not aware of any previous evidence that being more agreeable might increase the risk of adverse health outcomes. We had no prior hypothesis as to the relation between agreeableness and risk of frailty, but in view of findings from a meta-analysis that greater disagreeableness and, in particular, greater hostility—one of the facets of this trait—are linked with increased mortality [62], we were surprised by the direction and strength of the association that we found once the variance agreeableness shared with the other four personality traits had been removed. For it to be interpretable as a genuine effect, there would need to be a substantive and plausible definition of the variance that has been partialled out. It seems likely that this apparently suppressed effect of agreeableness is a statistical artefact.

The strengths of our study include the large sample size and the fact that it is representative of the community-dwelling English population aged 60 and over. Furthermore, it is the first investigation of the prospective relationship between personality and risk of frailty. The study also has some weaknesses. Firstly, scores for four of the five personality traits examined were moderately highly correlated (rho 0.41 to 0.59), which is not ideal in a personality inventory. We dealt with this by adjusting each trait for the other four traits in our general linear models. Secondly, data on BMI was not available at baseline, so we were unable to examine its potential mediating role in the associations between personality traits and later frailty. Thirdly, those who were excluded from our analytical sample due to loss to follow-up or missing data were older and frailer and had slightly lower scores for some personality traits than those who were included. However, all data were weighted to correct for non-response and for differential sample loss between waves. Finally, personality was measured when participants were aged 60 to over 90 years. Once people reach around age 30 years, personality traits tend to show considerable stability over time [63], but there is some evidence for change in personality in old age, perhaps in response to critical life events or major chronic illness [64]. It is possible that the presence of illness and disability at the time of personality assessment may have influenced how our participants responded when asked how much each adjective in the personality inventory applied to them. This may apply particularly to the adjectives active, lively and hardworking, part of the extraversion and conscientiousness scales, respectively. Participants’ self-ratings of these adjectives were much more strongly correlated with the contemporaneous frailty index score than were their ratings of other adjectives. In our analyses, we adjusted for frailty index score at the time of personality assessment, thereby taking account of all comorbidities, functional and sensory impairments and symptoms recorded at that time. Furthermore, a sensitivity analysis in the subset of participants whose frailty index score at baseline suggested that they had few or no problems produced estimates for the associations between personality traits and frailty at follow-up that were very similar to those obtained in the sample as a whole. However, when re-ran our analyses using modified extraversion and conscientiousness scores which had been calculated without using the extraversion items active, lively or the conscientiousness item hardworking, the association between extraversion and frailty at follow-up was no longer significant, although that between conscientiousness and later frailty persisted. This suggests that reverse causality may explain our observation linking lower extraversion to greater frailty at follow-up, though another interpretation could be that active and lively reflect the aspects of extraversion that are protective against frailty [65]. Our findings on the potential importance of certain personality traits as risk factors for the onset or progression of frailty need replicating, ideally in a cohort with a measure of personality taken much earlier in adult life.

In this prospective study of men and women aged 60 to over 90 years, higher levels of neuroticism and lower levels of conscientiousness and extraversion were associated with greater frailty around 2 years later. These associations were only slightly attenuated by adjustment for potential confounding or mediating factors, but there was some evidence that reverse causation may explain the link between extraversion and frailty risk. Future studies need to replicate our observations in other populations and explore the mechanisms whereby these personality traits might increase the risk of frailty.
